# Supplementary material for: Objective Methods of 5-Aminolevulinic Acid-Based Endoscopic Photodynamic Diagnosis Using Artificial Intelligence for Identification of Gastric Tumors
Source: J Clin Med. 2022 May 27;11(11):3030. doi: 10.3390/jcm11113030 (PMC9181250; doi:10.3390/jcm11113030)
Supplement: Supplementary file 1 [file jcm-11-03030-s001.zip › jcm-1695380-supplementary.pdf]

Supplementary Materials

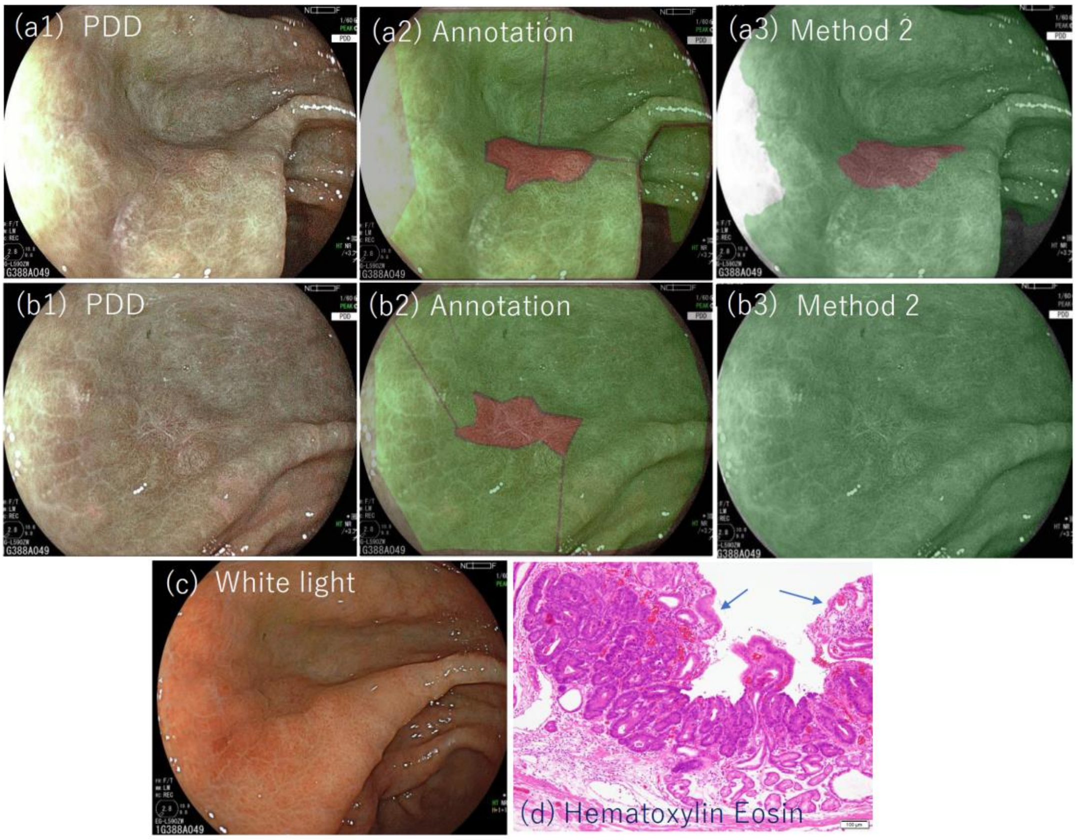

Supplementary Figure S1. Example case (lesion #4) with low area efficiency: (a1, b1) Photodynamic diagnosis images of lesion #4 (trained lesion); (a2) annotated image overlaid on (a1); (a3) the result of method two overlaid on (a1); (b2) annotated image overlaid on (b1); (b3) the result of method two overlaid on (b1); (c) white light image of lesion 4; (d) microscopic picture of hematoxylin and eosin staining of lesion #4. The blue arrow shows the surface non-tumor glands.

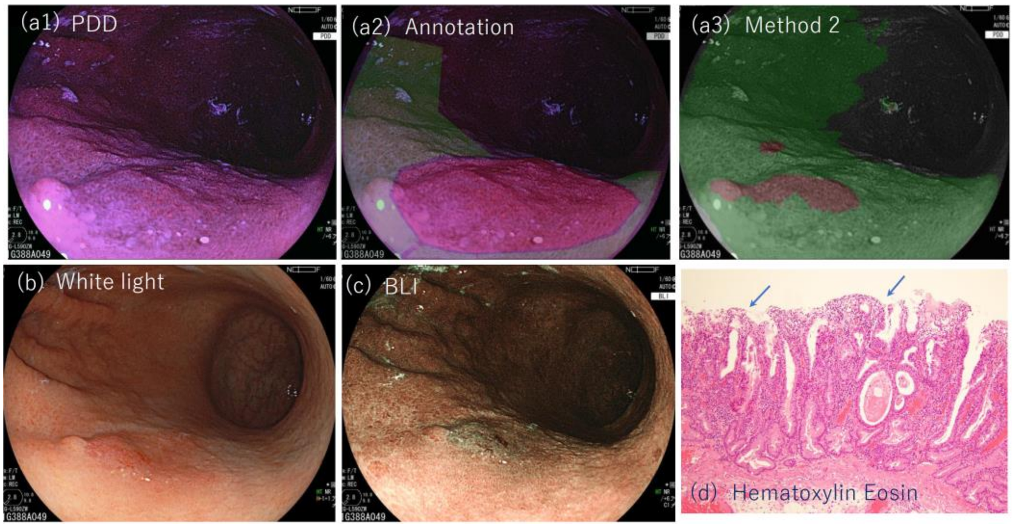

Supplementary Figure S2. Example case (lesion #18) with low area efficiency: (a1) Photodynamic diagnosis image of lesion #18 (validated lesion); (a2) annotated image overlaid on (a1), where tumor region is depicted in violet and non-tumor region is depicted in pink; (a3) the result of method two overlaid on (a1); (b) white light image of lesion #18; (d) microscopic picture of hematoxylin and eosin staining of lesion #18. The blue arrow shows the surface non-tumor glands.

Supplementary Table S1. Characteristics of the lesions included in this study. Morphology, size, histological diagnosis before resection (biopsy) and final histological diagnosis are shown. "Laser" or "LED" in the "Laser/LED" column shows that PDD is performed with laser-based system or LED-based system, respectively. Circle in the "Method one" column shows that method one was applied to the lesion. "Training" or "Validation" in "Method two Training/Validation" column shows that the lesion was used for training or validation in method two. Circle in the "Newly identified lesions" column shows that the lesion was first identified as tumor in PDD. Lesion #3, #21; Lesion #5, #25; Lesion #9, #22, #23; Lesion #10, #26; Lesion #16, #27; Lesion #20, #24 are from the same case, respectively. * Part of the lesion #3 was used in the analysis due to oozing (the original size of the lesion #3 is 29x18mm).

** One lesion of case #3, the original lesion planned to be resected by ESD, was excluded from the study because of oozing from the lesion.

Lesion #	Case #	Morphology	Size	Histological Diagnosis before resection	Final Histological Diagnosis (ESD or surgical(#20,#24) resection)	Laser/LED	Method one	Method two Training/ Validation	Newly identified lesion
**1**	1	0-IIa	19x13mm	tub1	tub1, M	Laser		Training	
**2**	2	0-IIc	18x16mm	tub2	tub1>tub2, M	Laser		Training	
**3**	3 **	0-IIb	10x9mm *	tub1	tub1, M	Laser		Training	○
**4**	4	0-IIc	7x7mm	tub1	tub1>tub2, M	Laser		Training	
**5**	5	0-IIc	30x25mm	tub1, tub2	tub1>tub2, M	Laser		Training	
**6**	6	0-IIa	12x10mm	tub1	tub1, M	Laser		Training	
**7**	7	0-IIc	20x15mm	Group4	tub1>tub2, SM1	LED	○	Training	
**8**	8	0-IIa	38x30mm	adenoma	tub1, M	LED	○	Training	
**9**	9	0-I	22x17mm	tub1	tub1> tub2>por, SM2	LED	○	Training	
**10**	10	IIc	30x21mm	tub1	adenoma	Laser		Training	
**11**	11	IIa	10x10mm	adenoma	adenoma	Laser		Training	
**12**	12	IIa	38x30mm	adenoma	adenoma	LED	○	Training	
**13**	13	0-IIc	17x12mm	tub1	tub1, M	Laser		Validation	
**14**	14	0-IIa	15x12mm	tub1	tub1+2>por, M	Laser		Validation	
**15**	15	0-I	40x34mm	tub1	tub1, SM2	Laser		Validation	
**16**	16	0-IIa	15x11mm	tub1 > tub2	tub1, M	Laser		Validation	
**17**	17	0-IIc	5x3mm	tub1	tub1, M	Laser		Validation	○
**18**	18	0-IIa	21x20mm	tub1	tub1, M	Laser		Validation	
**19**	19	0-IIb	21x11mm	tub1	tub1, M	LED	○	Validation	
**20**	20	0-IIa	10x6mm	tub1	tub1, M	LED	○	Validation	
**21**	3 **	0-IIb	10x10mm	tub1	tub1, M	Laser		Validation	○
**22**	9	0-IIc	25x15mm	tub1	tub1>tub2, M	LED	○	Validation	
**23**	9	0-IIc	13x10mm	tub1	tub1, M	LED	○	Validation	
**24**	20	0-IIa+IIc	12x10mm	EBV-related	EBV-related, SM2	LED	○	Validation	
**25**	5	IIa	30x20mm	adenoma	adenoma	Laser		Validation	
**26**	10	IIa+IIb	30x25mm	adenoma	adenoma	Laser		Validation	
**27**	16	IIa	16x8mm	adenoma	adenoma	Laser		Validation	
